# Analysis of biogenic carbonyl compounds in rainwater by stir bar sorptive extraction technique with chemical derivatization and gas chromatography‐mass spectrometry

**DOI:** 10.1002/jssc.201600561

**Published:** 2016-12-29

**Authors:** Xiaobing Pang, Alastair C. Lewis, Marvin D. Shaw

**Affiliations:** ^1^ Key Laboratory for Aerosol‐Cloud‐Precipitation of China Meteorological AdministrationNanjing University of Information Science and TechnologyNanjingChina; ^2^ Wolfson Atmospheric Chemistry LaboratoriesDepartment of ChemistryUniversity of YorkYorkUK; ^3^ National Centre for Atmospheric ScienceUniversity of YorkYorkUK

**Keywords:** biogenic carbonyl compounds, rainwater, stir bar sorptive extraction, volatile organic compounds

## Abstract

Stir bar sorptive extraction is a powerful technique for the extraction and analysis of organic compounds in aqueous matrices. Carbonyl compounds are ubiquitous components in rainwater, however, it is a major challenge to accurately identify and sensitively quantify carbonyls from rainwater due to the complex matrix. A stir bar sorptive extraction technique was developed to efficiently extract carbonyls from aqueous samples following chemical derivatization by *O*‐(2,3,4,5,6‐pentafluorobenzyl) hydroxylamine hydrochloride. Several commercial stir bars in two sizes were used to simultaneously measure 29 carbonyls in aqueous samples with detection by gas chromatography with mass spectrometry. A 100 mL aqueous sample was extracted by stir bars and the analytes on stir bars were desorbed into a 2 mL solvent solution in an ultrasonic bath. The preconcentration Coefficient for different carbonyls varied between 30 and 45 times. The limits of detection of stir bar sorptive extraction with gas chromatography mass spectrometry for carbonyls (10–30 ng/L) were improved by ten times compared with other methods such as gas chromatography with electron capture detection and stir bar sorptive extraction with high‐performance liquid chromatography and mass spectrometry. The technique was used to determine carbonyls in rainwater samples collected in York, UK, and 20 carbonyl species were quantified including glyoxal, methylglyoxal, isobutenal, 2‐hydroxy ethanal.

**Abbreviations**
CCN: cloud condensation nuclei
ECD: electron capture detection
GLY: glyoxal
MGLY: methylglyoxal
PDMS: polydimethylsiloxane
PFBHA: *O*‐(2,3,4,5,6‐pentafluorobenzyl) hydroxylamine
SBSE: stir bar sorptive extraction
SOA: secondary organic aerosol
VOC: volatile organic compound

## 1 INTRODUCTION

Carbonyl compounds (carbonyls) have been recognized to play a crucial role in atmospheric chemistry 1. Some small carbonyl compounds react with ammonium sulfate or amines to form light‐absorbing brown carbon in aqueous aerosol, which potentially influences global radiative forcing 2. Some highly water‐soluble carbonyl compounds such glyoxal (GLY) and methylglyoxal (MGLY) can form secondary organic aerosol (SOA) through uptake into the aqueous phase of an aerosol particle and form low‐volatility organonitrogen/organosulfate/oligomeric products 3. In cloud water some carbonyls such as formaldehyde, ethanal, acetone and propanal have been considered to be the precursors to SOA formation through cloud processing, which may alter the ability of cloud‐processed particles to act as cloud condensation nuclei (CCN) 1. In tropospheric aerosols, MGLY and ethanal were found to enhance aerosol CCN activity through depressing surface tension, contributing solute, and influencing droplet activation kinetics 4. Since GLY, MGLY, and some other carbonyls are common products from biogenic isoprene photooxidation and participate directly in the cloud processing, their concentrations and temporal variations in rainwater can be considered as one important marker for biogenic volatile organic compounds (VOCs) influence on cloud processing 3. Tools to measure carbonyl compounds in rainwater are therefore essential to improve knowledge of how the biogenic VOCs influence cloud droplet formation

The analysis of carbonyls in rainwater remains a challenge in analytical chemistry since most carbonyls in rainwater are volatile and present in trace levels. Chemical derivatization with 2,4‐dinitrophenylhydrazine and HPLC–MS analysis is a specific analytical method for aqueous carbonyls. Although such a method provides good reproducibility, the methods are less sensitive and present a lower‐resolution separation than GC analysis 5. Some trace carbonyls from biogenic VOCs photooxidation are not detected by HPLC analysis due to sensitivity issues, which leads to an underestimation of the biogenic contribution to atmospheric oxidization capacity 6. *O*‐(2,3,4,5,6‐Pentafluorobenzyl) hydroxylamine (PFBHA) is an effective chemical derivatization reagent for carbonyls and PFBHA‐oxime derivatives can be produced in high yields, which are amenable to GC–MS 7 and GC with electron capture detection (ECD). PFBHA is especially suitable for the derivatization of aqueous carbonyls since PFBHA is a hydrophilic reagent. One obstacle for the direct measurements of PFBHA‐oxime derivatives in rainwater by GC is that direct injection of an aqueous sample into GC column will damage the column and deteriorate the analytical reproducibility. Some sample treatment techniques have been employed to extract PFBHA‐oxime derivatives from aqueous bulk including SPE 8, SPME 9, 10, and stir bar sorptive extraction (SBSE) 11. The working principles of SPME and SBSE rely on analyte absorption (partitioning) on an absorption phase such as polydimethylsiloxane (PDMS) from the sample, whereas SPE is based on the analyte adsorption on the solid‐phase sorbent. The applications of above three pretreatment techniques are similar and mainly include the extraction of target analytes from water matrix by solid‐phase sorbent, solvent elution of analytes from sorbent, and then GC analysis. SPME techniques have some limitations such as the fragile nature of the fiber and the limited extraction capacity due to the small PDMS volume (0.5 μL) coated on fiber 12. SBSE technique has typically a higher absorption capacity and higher analytical recovery due to higher PDMS volumes compared with SPME 13. In comparison to SPE techniques, the processes of SBSE application are less complicated including the extraction and the desorption processes. It should be noted that the SPE technique has a higher sorption capacity compared to SBSE techniques but that SPME technique is superior in the simplicity of the procedure.

SBSE is based on analyte absorption (partitioning) from the sample solution onto a PDMS film coated onto a glass‐coated magnetic stir bar 13. Four size stir bars are commercially provided by Gerstel, Mülheim an der Ruhr (Germany) with PDMS volumes of 24, 47, 63, and 126 μL, respectively. The higher volumes of PDMS coated on SBSE compared to that of PDMS on SPME (0.5 μL) increase the amount of analyte extracted from the sample solution for the target analytes with low partition coefficients such as carbonyl PFBHA derivatives. SBSE technique is usually combined with a thermal desorption but a solvent desorption is preferable giving the possibility for replicate analysis and GC or HPLC combination 14, 15. SBSE technique was first applied to trace analysis of volatile compounds in gaseous samples 16 and later extended to the extraction of polycyclic aromatic hydrocarbons and some VOCs in environmental aqueous samples 17, 18, 19, 20.

In this study, SBSE technique was deployed to preconcentrate 29 carbonyl species in the aqueous phase after being derivatized by PFBHA. The newly developed technique was employed to measure the temporal variation of carbonyls in rainwater collected in York, UK. The environmental implications of biogenic carbonyls in rainwater are discussed in respect of CCN functions.

## 2 MATERIALS AND METHODS

### 2.1 Reagents and apparatus

All chemicals including 29 species of carbonyl compound, whose properties are listed in Table 1, PFBHA (99%), humic acid, acetonitrile (ACN, HPLC grade) were purchased from Sigma–Aldrich (Gillingham, UK). Two commercial sizes of stir bars (Twister®) coated by PDMS (63 and 126 μL) were obtained from Gerstel. Two identical magnetic stirrers (RH Basic 2 IKAMAG, UK) were employed to twist the stir bar directly in aqueous solution.

**Table 1:** Peak identifications of carbonyl‐PFBHA derivatives in GC–MS chromatogram shown in Fig. 1 and molecular weights of carbonyls and their derivatives

No.	Carbonyls	Peaks	RT (min)	Linear formula	MW	Derivative MW
1	Formaldehyde	1	2.50	HCHO	30	225
2	Ethanal	2, 3a)	2.99, 3.03	CH_3_CHO	44	239
3	Acetone	4	3.30	CH_3_COCH_3_	58	253
4	Propanal	5, 6	3.40, 3.43	CH_3_CH_2_CHO	58	253
5	Propenal	7	3.51	CH_2_ = CHCHO	56	251
6	Butanal	8	3.59	CH_3_(CH_2_)_2_CHO	72	267
7	Isobutenal	9	3.75	CH_3_CH = CHCHO	70	265
8	Butenone	10	3.78	CH_3_COCH = CH_2_	70	265
9	2‐Hydroxy ethanal	11, 12	3.82, 3.87	HOCH_2_CHO	60	255
10	2‐Pentanone	13	4.01	CH_3_CO(CH_2_)_2_CH_3_	86	281
11	3‐Methylbutanal	14, 15	4.06, 4.09	C_2_H_5_CH(CH_3_)CHO	86	281
12	2‐Butenal	16, 17	4.14, 4.18	CH_3_CH = CHCHO	70	265
13	Pentanal	18, 19	4.25, 4.27	CH_3_(CH_2_)_3_CHO	86	281
14	2‐Hexanone	20, 21	4.37, 4.42	CH_3_(CH_2_)_3_COCH_3_	100	295
15	Hydroxyacetone	23, 24	4.52, 4.60	CH_3_COCH_2_OH	74	269
16	Pentane‐2,4‐dione	22	4.47	CH_3_COCH2COCH3	100	295
17	Hexanal	25, 26	4.66, 4.68	CH_3_(CH_2_)_4_CHO	100	295
18	2‐Hexenal	27, 28	4.75, 4.81	CH_3_(CH_2_)_2_CH = CHCHO	98	293
19	Heptanal	29, 30	4.90, 4.92	CH_3_(CH_2_)_5_CHO	114	309
20	2‐Octanone	31, 32	5.05, 5.09	CH_3_(CH_2_)_5_COCH_3_	128	323
21	Octanal	33, 34	5.19, 5.27	CH_3_(CH_2_)_6_CHO	128	323
22	4‐Fluorobenzaldehyde	35	5.42	FC_6_H_4_CHO	124	319
23	Benzaldehyde	36, 37	5.49, 5.53	C_6_H_5_CHO	106	301
24	*o*‐Tolualdehyde	38, 39	5.56, 5.63	CH_3_C_6_H_4_CHO	120	315
25	*m*‐Tolualdehyde	40, 41	5.69, 5.84	CH_3_C_6_H_4_CHO	120	315
26	*p*‐Tolualdehyde	42, 43	5.91, 5.93	CH_3_C_6_H_4_CHO	120	315
27	2,4‐Bimethylbenzaldehyde	44, 45	5.97, 6.16	(CH_3_)_2_C_6_H_3_CHO	134	329
28	GLY	46, 47, 48	6.25, 6.40, 6.44	OHCCHO	58	448
29	MGLY	48, 49	6.51, 6.57	OCCH_3_CHO	74	462

a) Two peaks/isomers are formed from asymmetric carbonyls.

### 2.2 Cleaning procedure

All the glassware and tweezers were first cleaned with abundant water and then rinsed with ultrapure water provided by a water purification equipment (ELGA‐PURELAB flex system, Veolia, France). The glassware and tweezers were dried in an oven at 250°C for 3 h to eliminate the possible interferences from the organics. Powder‐free nitrile examination gloves approved for medical use (Microflex 93–843, Ansell) were employed in the conductions to avoid contaminations during the whole experiment. Before use each stir bar was put in a glass vial containing 10 mL acetonitrile and cleaned in an ultrasonic bath for 30 min to desorb all organics on the bar.

### 2.3 Derivatization and SBSE procedures

A 100 mL rainwater or other aqueous sample was poured into a glass bottle (200 mL size) and 1 mL PFBHA solution (1 mg/mL) was added simultaneously. The aqueous solution in the bottle was adjusted to pH 6.0 by 3 mL NaH_2_PO_4_‐HCl buffer solution and left overnight (14 h) for the completeness of derivatization reaction between carbonyls and PFBHA 21. The stir bar was put into the glass bottle containing 105 mL sample solution after the addition of 1 mL NaCl solution, which was located on a magnetic stirring plate. The stir bar was employed to extract carbonyl derivatives at speed of 500 rpm for 1 h. After extraction the stir bar was removed from the sample solution and dried by a tissue paper to clean the solution adhering to its surface. The stir bar was transferred into 2.0 mL acetonitrile as desorption solvent a 10 mL vial and desorbed by ultrasonic bath for 30 min. One microliter of the remaining acetonitrile solution was directly injected into a Perkin Elmer GC–MS (Waltham, USA) for analysis. The stir bar was cleaned as described above and stored in acetonitrile for future use. During the optimization process, several important SBSE parameters such as extraction time (30 min to 16 h), ultrasonic desorption time (10 min to 3 h), desorption solvents (acetonitrile, ethanol, methanol) and the temperatures of extraction solution were optimized.

### 2.4 Standard solutions for calibration

To obtain the calibration curve for each analyte, seven standard solutions of carbonyl‐PFBHA derivatives varying from 0.5 to 60 μg/L were prepared from the dilutions of a stock solution (200 μg/L for each carbonyl‐PFBHA derivative). In each standard solution 4‐fluorobenzaldehyde was added as internal standard with its concentration at 10 μg/L. The stock solution was prepared by adding 150 mg PFBHA into 1 L solution containing 29 species of carbonyls at 200 μg/L. The excess of PFBHA to carbonyl derivatizations was favorable for the formation of carbonyl derivatives in high yield near to 100%. The calibration curves were established by plotting the peak areas (sum of *E* and *Z* isomers) versus the carbonyl derivative concentrations from 0.5 to 60 μg/L. The slope of calibration curves, linear ranges, and the correlation coefficients (*r* ^2^) are listed in Table 2. The LODs were calculated experimentally by spiking ultrapure water with carbonyl derivatives at concentration levels close to the theoretical LODs, of which signals are three times greater than the baseline noise. The LODs that were obtained using spiked ultrapure water may be a little different to those of the rainwater samples due to a lower matrix effect in ultrapure water but they provide a guideline for sensitivity of the method.

**Table 2:** Slopes and coefficients (*r* ^2^) of calibration curves, linear ranges, LODs, repeatability, and reproducibility of the optimized SBSE technique for aqueous carbonyl analysis

			Linear rangeb)		LOD	Recoveryc)	Repeatabilityd)	Reproducibilitye)
No.	Carbonyls	Slopea)	(μg/L)	*r^2^*	(ng/L)	(%, *n* = 5)	(%)	(%)
1	Formaldehyde	381.1	0.02–28	0.986	14	103	6.6	7.8
2	Ethanal	345.4	0.04–32	0.971	31	98.2	7.4	10.2
3	Acetone	181.3	0.06–36	0.968	57	106	8.5	14.3
4	Propanal	384.5	0.03–36	0.975	27	86.3	7.6	8.5
5	Propenal	236.1	0.04–36	0.966	33	83.4	12.3	12.2
6	Butanal	270.4	0.03–36	0.962	28	90.2	5.3	7.6
7	Isobutenal	33.5	0.27–48	0.985	266	88.3	12.7	14.7
8	Butenone	41.3	0.19–44	0.984	189	81.4	11.8	12.8
9	2‐Hydroxy ethanal	65.8	0.18–40	0.971	178	80.3	8.4	12.5
10	2‐Pentanone	102.2	0.08–40	0.949	76	90.3	5.3	13.6
11	3‐Methylbutanal	285.7	0.06–36	0.982	57	92.4	6.9	8.9
12	2‐Butenal	340.1	0.05–32	0.973	42	79.3	9.4	12.5
13	Pentanal	362.1	0.03–32	0.984	21	86.4	5.8	11.5
14	2‐Hexanone	84.7	0.12–40	0.983	112	88.3	7.3	11.6
15	Hydroxyacetone	63.9	0.13–40	0.984	122	90.4	8.4	10.6
16	Pentane‐2,4‐dione	57.5	0.24–44	0.973	236	92.4	9.4	12.6
17	Hexanal	247.3	0.04–36	0.972	32	90.4	11.3	11.5
18	2‐Hexenal	165.4	0.05–36	0.974	47	87.4	13.6	12.1
19	Heptanal	248.6	0.04–32	0.982	31	93.6	14.6	13.6
20	2‐Octanone	39.4	0.2–44	0.983	198	92.3	9.5	11.5
21	Octanal	248.6	0.04–32	0.974	31	90.4	7.9	11.6
22	4‐Fluorobenzaldehyde	268.4	0.06–32	0.966	59	97.0	4.8	8.5
23	Benzaldehyde	305.3	0.05–32	0.972	46	94.5	5.5	9.2
24	*o*‐Tolualdehyde	187.5	0.05–32	0.964	42	104	6.7	8.6
25	*m*‐Tolualdehyde	208.7	0.07–32	0.964	67	105	7.5	6.2
26	*p*‐Tolualdehyde	229.4	0.09–32	0.973	84	95.6	9.6	9.6
27	2,4‐Bimethylbenzaldehyde	246.2	0.08–32	0.973	72	95.3	8.3	10.2
28	GLY	375.5	0.02–28	0.985	16	89.5	11.9	10.3
29	MGLY	338.4	0.02–28	0.986	18	94.8	13.9	12.5

a) Peak area = Slope × *c* (Unit for *C*: μg/L).

b) Data are obtained from seven to ten aqueous calibration solutions.

c) Mean of five determinations.

d) Mean of five determinations.

e) Mean of 15 determinations (five determinations each day for 3 days).

### 2.5 Repeatability and reproducibility

To illustrate the SBSE technique capability, the repeatability of SBSE technique was evaluated by determining concentrations in five ultrapure water samples spiked with 8 μg/L of each carbonyl at the same day. Reproducibility was calculated through the measurements of above water samples in three different days. The repeatability and reproducibility were expressed as the RSD = [(SD of observed concentration)/(average of observed concentration)] ×100%.

### 2.6 Matrix effects and recovery

To investigate the matrix effects of others chemicals in natural water, an artificial rainwater sample was prepared by the addition of the typical chemical compositions in rainwater including K^+^, Na^+^, NH_4_^+^, Ca^2+^, Mg^2+^, Cl^−^, NO_3_^−^, SO_4_^2–^ and humic acid. The concentrations of those ions and humic acid are 1–2 mg/L. Then an amount of stock solution of carbonyls was spiked into the artificial rainwater and the concentrations of carbonyls maintained at 8 μg/L. The observed concentrations of the carbonyls in five parallel artificial rainwater samples were quantified by SBSE technique under the optimal conditions. The recoveries of carbonyls were calculated by the equation of [observed concentration/absolute concentration] ×100% to evaluate the matrix effects of chemical composition on SBSE technique.

### 2.7 Rainwater Sampling

Five rainwater samples were collected in the campus of University of York, York, UK from July to November 2014. The detailed information of the rainfall samples is shown in Table 3. After collection the rainwater samples were immediately derivatized by PFBHA and treated by the SBSE procedures, which were conducted under the optimal conditions described earlier. York (53°57’30”N, 1°4’49”W) is located in the north of England, with an urban population of about 150 000. No significant industrial emissions are observed near York since the city economy is dominantly based on tourism, health, and education. The city covers a great amount of deciduous woods mainly including English Oak (*Quercus robur*), European Ash (*Fraxinus excelsior*), European Beech (*Fagus sylvatica*), Sliver Birch (*Betula pendula*), Horse Chestnut (*Aesculus hippocastanum*), *etc*.

**Table 3:** Detailed information of rainwater collected from July to November 2014 in York, UK

	Jul.	Aug.	Sep.	Oct.	Nov.
Date of rain	19	8	29	5–6	7–8
Rainfall amount (mm)	13.8	50.6	26.4	33.6	22.5
Time interval from last rain (day)	6	19	21	5	30
Rain duration (h:m)	3:08	6:20	3:52	5:50	4:35
pH	5.4	6.8	4.9	7.6	7.4

### 2.8 GC–MS conditions

Separation and detection of the carbonyl‐PFBHA derivatives were performed on a Perkin Elmer GC–MS incorporating an Auto system XLGC and a quardrupole MS equipped with a DB5 column (30 m × 0.25 mm × 1.0 μm) (Agilent Technologies, Santa Clara, USA). GC conditions were as follows: the GC oven temperature was initially set at 80°C for 1 min, programmatically increased to 260°C with a ramp of 30°C min^−1^. The solvent delay was set at 2 min. Helium (CP Grade (N5.0) 99.999%, BOC, Guildford, UK) was chosen as the carrier gas at a flow rate of 1 mL/min. The GC injection mode was set as splitless. The temperatures of GC inlet and GC–MS transfer line were kept at 250°C. The mass spectrometer was operated in scan mode with a mass range of 100–500 Da to identify the most abundant ions of carbonyl derivatives. The chromatograms at the most abundant ion were used to quantify the concentration of derivatives in solution. In this study the most abundant ions of all carbonyl‐PFBHA derivatives are the same as ([C_6_F_5_CH_2_•]^+^) with *m/z* = 181 Da.

## 3 RESULTS AND DISCUSSIONS

### 3.1 Theory of SBSE

The extraction efficiency of SBSE in PDMS stationary phases is correlated to the octanol‐water partitioning coefficient (*K*_o/w_) of analyte and to the phase ratio (β) of sample. The equations that guide the partition between the liquid and the stationary phases are

(1) m SBSE mo=Ko/wKo/wββ1+Ko/wKo/wββ

(2) β= Volume  of  sample  Volume  of  stationary  phase

where *m*_SBSE_ is the mass of analyte in the sorbent and *m*_o_ is the mass of the analyte in solution. For a specific sample in a known volume with a stable value for *β*, the extraction efficiencies of an analyte are positively correlated with the *K*_o/w_ values, which are constant to specific compounds 22. Nonetheless, equation (E1) is only valid when the equilibrium has been reached and it is difficult to reach the theoretical extraction efficiency in equation (E1) for a complex solution containing several analytes due to their different equilibrium times. Therefore, extractions under equilibrium conditions are not always possible and compromise conditions have to be set. For the SBSE technique, extraction time, stirring rate, pH value, ionic strength, desorption solvent, and desorption time can affect the equilibrium and extraction efficiency. To achieve optimal extraction efficiency, the above factors have been investigated and optimized based on the performances in the dominant carbonyls in rainwater including GLY, MGLY, formaldehyde, ethanal, acetone, 2‐hydroxy ethanal, and isobutenal.

### 3.2 Optimization of extraction conditions

SBSE is an equilibrium technique based on the partitioning of the analytes between the aqueous phase and the PDMS phase. Extraction time, stirring rate, pH value, ionic strength, desorption solvent, and desorption time are the important factors that can affect the equilibrium and efficiency of both extraction and desorption. To achieve optimal SBSE performance the above factors were investigated and optimized in this study.

#### 3.2.1 Effect of PDMS volume

**Figure 1:**
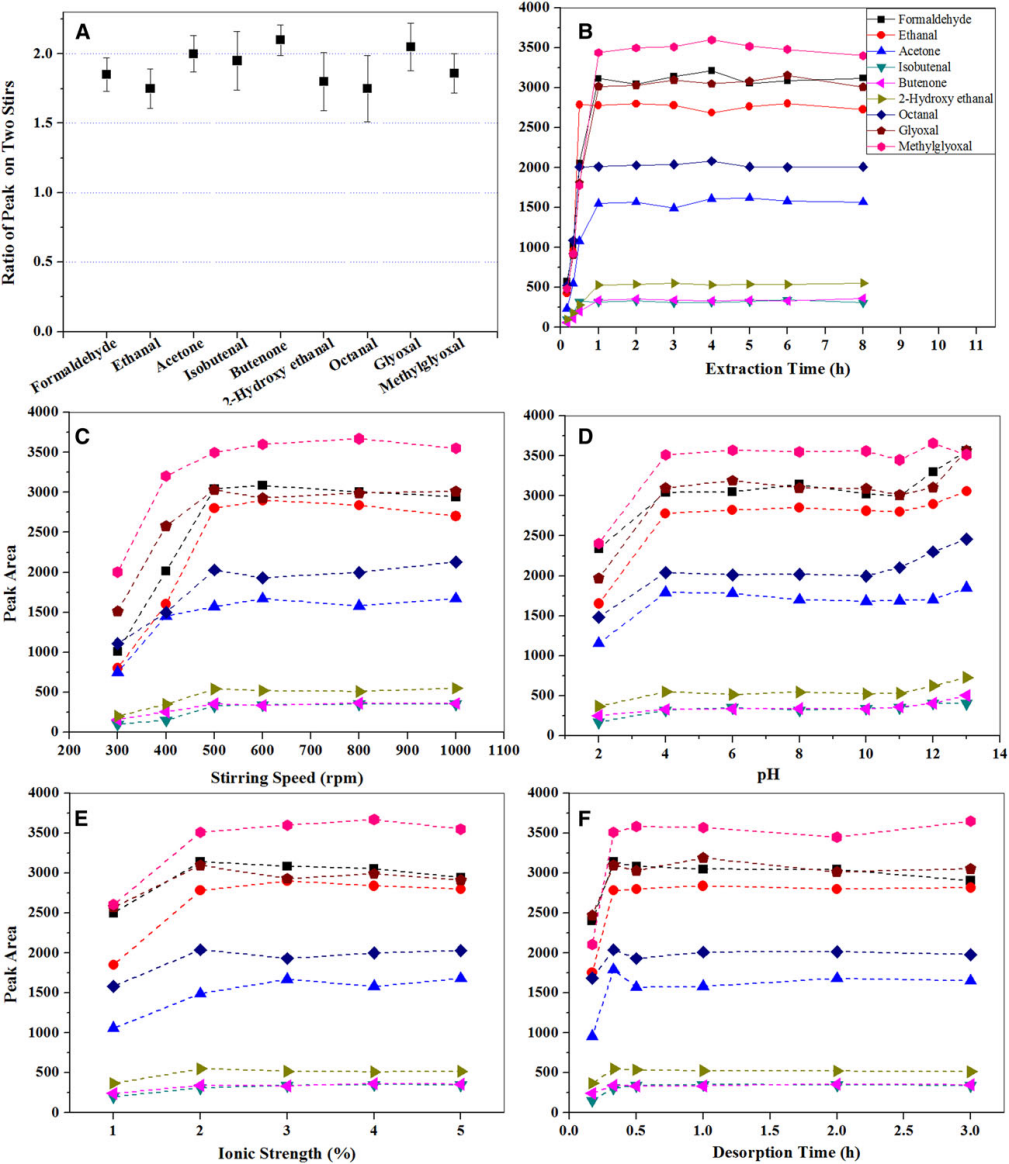
Effects of stir bar size (panel **A**), extraction time (panel **B**), stirring rate (panel **C**), pH of aqueous solution (panel **D**), ionic strength (panel **E**), desorption time (panel **F**) on SBSE technique employment. Nine carbonyls at 8 μg/L in 100 mL aqueous solution were investigated by the SBSE technique after chemical derivatization by PFBHA including formaldehyde, ethanal, acetone, isobutenal, butenone, 2‐hydroxy ethanal, octanal, GLY, and MGLY, respectively. Two sizes of stir bar coated with 63 and 126 μL PDMS were studied in the experiment of panel **A**

Two sizes of stir bar coated with 63 and 126 μL PDMS were investigated to evaluate the extraction efficiency. It was found that the concentrations of carbonyl derivatives desorbed from stir bar were with 126 μL PDMS were around two times higher than those from stir bar with 63 μL PDMS (Fig. 1A). This phenomenon was consistent with the manufacturer description that the extraction capacity of stir bar is proportional to the volume of PDMS. The stir bar with 126 μL PDMS was chosen in this study.

#### 3.2.2 Effect of extraction time

The effect of extraction time varied over the ranges of 10 min to 8 h on the extraction efficiency of target carbonyls was investigated. Experimental results showed that extraction equilibrium for all carbonyls was almost reached after 1 h extraction (Fig. 1B). Therefore, the extraction time of 1 h was chosen for the following experiments.

#### 3.2.3 Effect of stirring rate

Stirring can accelerate molecular mass transfer rate between PDMS layer and aqueous solution and further reduce the time for thermodynamic equilibrium. The influence of stirring rate varied in the range of 300–1000 rpm on the SBSE extraction efficiency was studied. The results indicated that the extraction efficiency increased with the stirring rate and then became stable after reaching the stirring rate of 500 rpm (Fig. 1C). Too high a stirring rate may damage the stir bars and a rate of 500 rpm was used in this work.

#### 3.2.4 Effect of pH value

Since the pH values of environmental samples such as rainwater, fog water, cloud water, and snow vary dramatically, it is essential to study the effect of the sample pH value on the SBSE performance. The pH values of solution varying in the range of pH 2.0–13.0 were studied. No significant differences of SBSE performance were observed in the range of pH 4.0–13.0 but with some slightly lower peak heights of carbonyl derivatives were found at pH 2.0 (Fig. 1D). From above results the pH effect on SBSE performance was negligible at most reasonable atmospheric conditions. In the following experiments, pH 6.0 was selected as the optimal pH value for SBSE extraction.

#### 3.2.5 Effect of ionic strength

The effect of ionic strength on the extraction was investigated and the experimental results are shown in Fig. 1E. The extraction efficiency of SBSE for all target carbonyls increased with the increase of NaCl concentration from 0 to 2% and then remained stable with further increases of NaCl concentration up to 5%. This phenomenon can be explained by two processes that occurred during the extraction process. Initially, the extraction efficiency for carbonyls increased with the increase of NaCl concentration due to the salt‐out effect, which drives more molecular carbonyl‐PFBHA derivatives into the PMDS layer. The further addition of NaCl increases the solution viscosity, which may potentially decrease the extraction efficiency 23. The extraction efficiencies remained constant over the range 2–5% salt concentration and a 2% NaCl in the sample solution was chosen as the optimal ionic strength in this work.

### 3.3 Effect of desorption solvent and desorption time

In this work, liquid desorption was employed rather than thermal desorption and an appropriate solvent should be chosen to achieve the highest possible desorption efficiency. Acetonitrile, ethanol, and methanol as desorption solvents were investigated. When methanol or ethanol was used as the desorption solution a pronounced peak at retention time of formaldehyde or acetaldehyde derivative was observed in the blank sample. The reason for above phenomena may be that traces of formaldehyde or acetaldehyde were present in the methanol or ethanol, respectively 24. Acetonitrile was selected as the optimal desorption solvent since it did not contain any carbonyl interferences.

The effect of desorption time varying from 10 min to 3 h on desorption efficiency was investigated (Fig. 1F). It was found that the desorption process reached close to desorption equilibrium after 20 min, and hence a conservative desorption time was set at 30 min in the study.

### 3.4 Effect of temperature on extraction and desorption

Temperature can affect the kinetics of the absorption of the molecules on the stir bar from aqueous solution and the desorption from the stir bar to the solvent 25. The effect of temperature on extraction and desorption were investigated under 20, 40, and 60°C. The equilibrium periods for the extraction process were 60, 50, and 20 min while those of desorption process were 20, 15, and 10 min, at 20, 40, and 60°C, respectively. The concentrations of extracted analytes remained approximately constant at three temperatures. Twenty degrees (lab ambient temperature) was chosen as the optimal temperature for extraction and desorption processes by SBSE. The optimal extraction conditions of SBSE technique for aqueous carbonyls were summarized as 60 min of extraction time, 30 min of liquid desorption time, pH 6.0 as the optimal pH, 2% NaCl as the optimal ionic strength, respectively. Acetonitrile was the desorption solvent. Stir bar works at 500 rpm at 20°C.

### 3.5 Performances and preconcentration efficiency of SBSE

**Figure 2:**
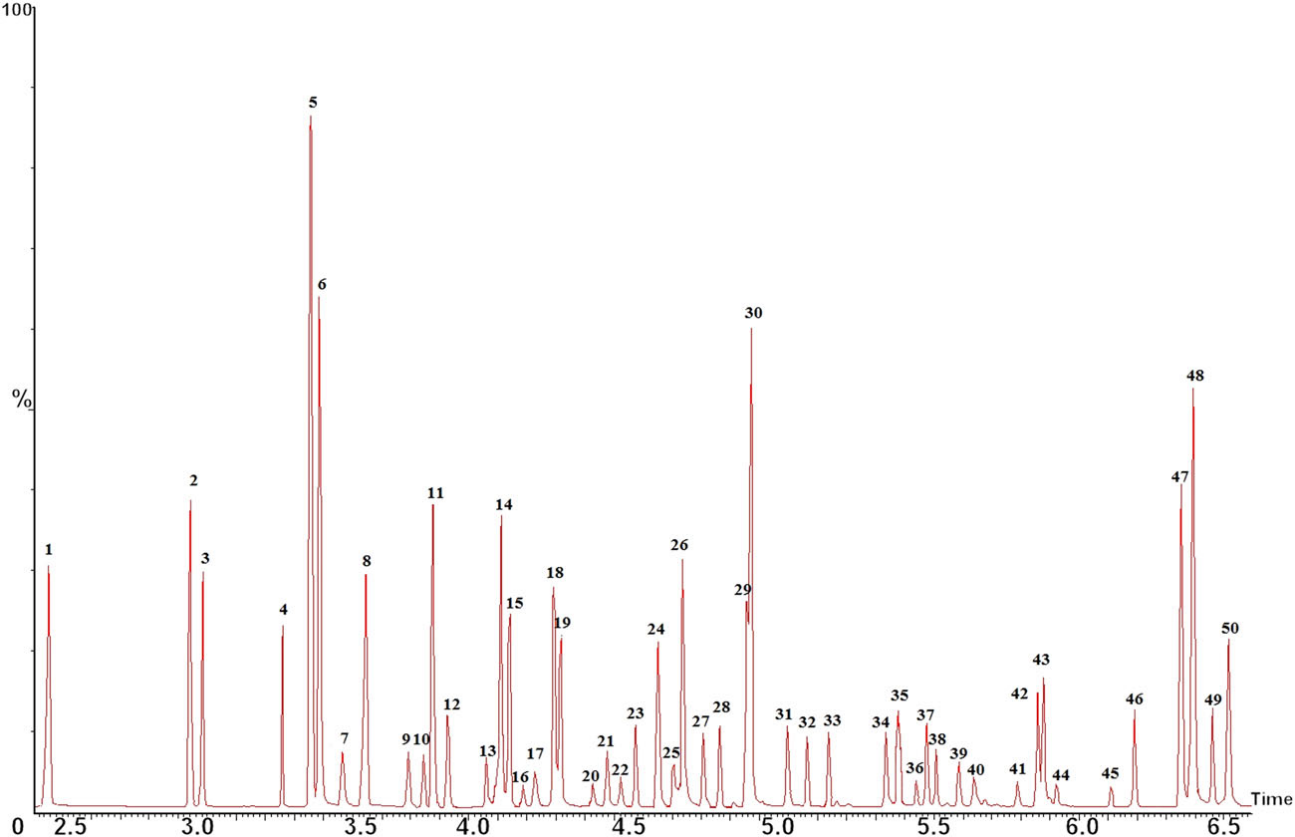
GC–MS chromatogram of 29 species of carbonyl‐PFBHA derivatives at 8 μg/L for most carbonyls except for propanal and heptanal at 16 μg/L, which were absorbed from 105 mL solution by stir bar and then desorbed into 1 mL acetonitrile from stir bar. Extraction and desorption processes were performed under optimal conditions by the SBSE. Identification of peaks corresponds to the same as listed in Table 1. Formaldehyde (peak 1), ethanal (peaks 2 and 3), acetone (peak 4), propanal (peaks 5 and 6), propenal (peak 7), butanal (peak 8), isobutenal (peak 9), butanone (peak 10), 2‐hydroxy ethanol (peaks 11 and 12), 2‐pentanone (peak 13), 3‐methylbutanal (peaks 14 and 15), 2‐butenal (peaks 16 and 17), pentanal (peaks 18 and 19), 2‐hexanaone (peaks 20 and 21), pentane‐2,4‐dione (peak 22), hydroxyactone (peaks 23 and 24), hexanal (peaks 25 and 26), 2‐hexenal (peaks 27 and 28), heptanal (peaks 29 and 30), 2‐octanone (peaks 31 and 32), octanal (peaks 33 and 34), 4‐fluorobenzaldehyde (peak 35), benzaldehyde (peaks 36 and 37), *o*‐tolualdehyde (peaks 38 and 39), *m*‐tolualdehyde (peaks 40 and 41), *p*‐tolualdehyde (peaks 42 and 43), 2,4‐bimethylbenzaldehyde (peaks 44 and 45), GLY (peaks 46, 47 and 48), MGLY (peaks 48 and 49), respectively. GC–MS conditions are described in the experimental Section 2

**Figure 3:**
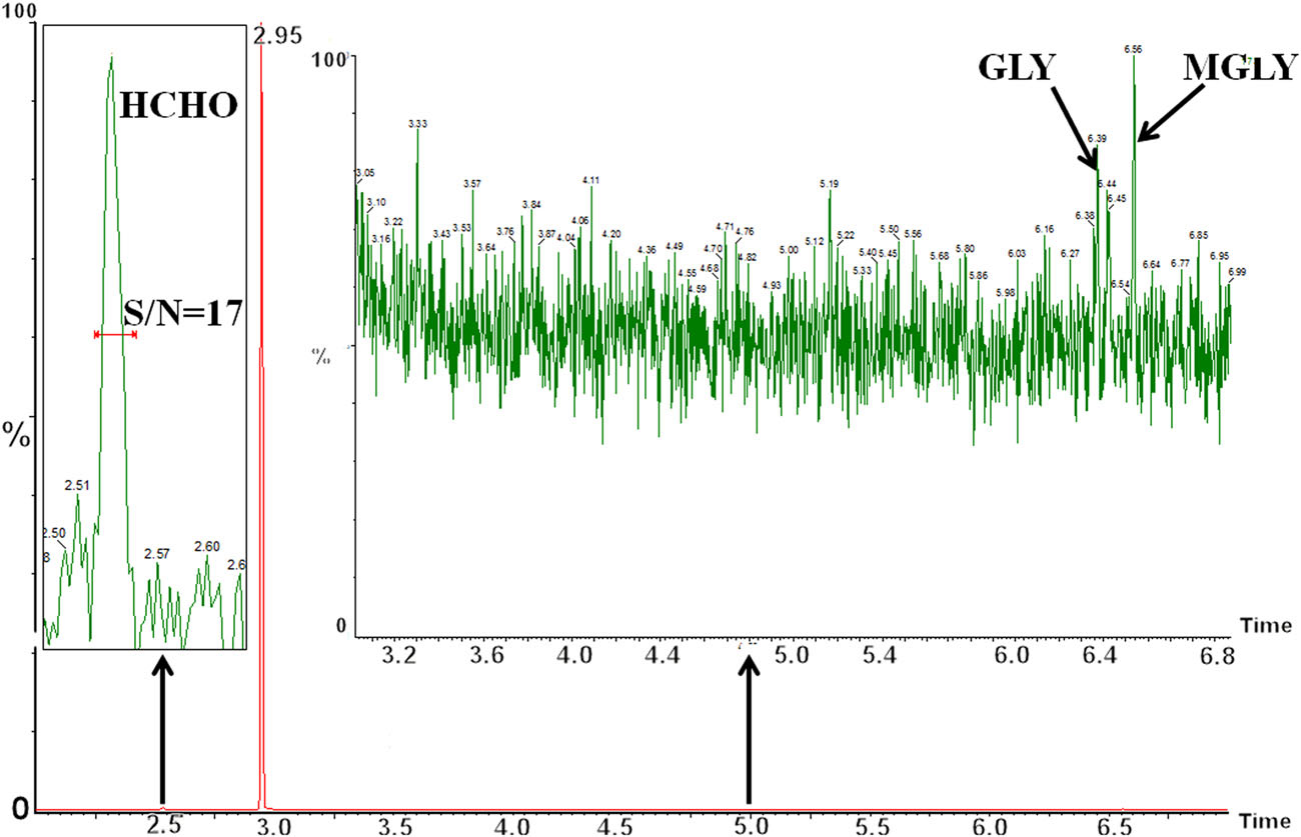
GC chromatogram of blank sample (lower panel) in solvent desorption solution after SBSE extraction. Traces of formaldehyde, GLY, and MGLY were observed in the blank sample. Insert panels are the enlargements of GC chromatogram of blank sample. Blank sample was prepared by using 100 mL ultrapure water mixed with 1 mL PFBHA solution (15 mg/mL), 3 mL buffer solution

**Figure 4:**
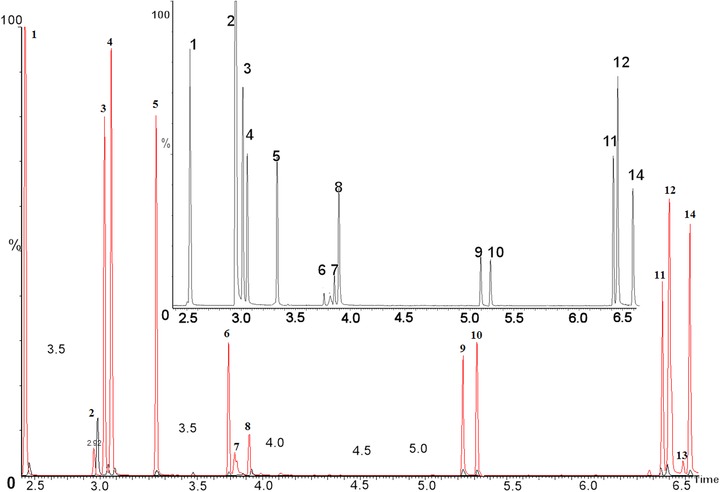
Overlay GC chromatograms of desorption solution containing nine carbonyl‐PFBHA derivatives (red line) and the solution before SBSE extraction (black line) to study preconcentration efficiencies of SBSE on formaldehyde (peak 1), ethanal (peaks 3 and 4), acetone (peak 5), isobutenal (peak 6), butenone (peak 7), 2‐hydroxy ethanal (peak 8), octanal (peaks 9 and 10), GLY (peaks 11 and 12), and MGLY (peaks 13 and 14), respectively. The inserted panel is the enlargement of GC chromatogram of carbonyl derivative solution before SBSE extraction (black line).

From the GC chromatogram of SBSE desorption solution in Fig. 2, 29 species of carbonyl‐PFBHA derivatives can be effectively separated from baseline. The retention time of the derivatives vary from 2.50 min (formaldehyde) to 6.57 min (MGLY) and the detailed retention times of each derivative are shown in Table 1. The derivatization reactions between carbonyls and PFBHA can be found in our previous studies 7, 24 and *E*/*Z* isomers can be formed by the derivatization of asymmetric carbonyls 26. For some dicarbonyls the *E* and *Z* isomerism occurs from derivative formation with both carbonyl groups, thus four isomers can be produced. The *E* and *Z* isomers cannot be chromatographically resolved in a few cases such as 2‐methylbutanal, nonanal, and decanal. A coelution between two isomers of GLY and MGLY also occurs. The sum of the isomer peak areas for each carbonyl derivative was used for quantification. The performance of SBSE for solvent and blank samples was essential to judge whether the SBSE technique would cause more contamination or not. In this study, the solvent (acetonitrile) and blank sample (mixture of 100 mL ultrapure water, 1 mL PFBHA solution (15 mg/mL), 3 mL buffer and 1 mL NaCl solutions) were tested by SBSE and their GC chromatograms implied no carbonyl derivatives present in SBSE desorption solution, but with trace formaldehyde, GLY, and MGLY observed in SBSE solution (Fig. 3). Hydrophobic PDMS on stir bar can extract efficiently hydrophobic analytes including carbonyl derivatives from the aqueous solution. The preconcentration efficiencies of SBSE were found to be different for the carbonyl derivatives at the same concentration in this study (Fig. 4). Nine carbonyl derivatives were chosen as target compounds including formaldehyde, ethanal, acetone, isobutenal, butenone, 2‐hydroxy ethanal, octanal, GLY, and MGLY, respectively. The preconcentration efficiency for each derivative was demonstrated as the ratio of each derivative's peak area in SBSE extraction solution to that in the initial aqueous solution, which is unitless. The higher preconcentration efficiencies were observed among the derivatives of octanal, GLY, MGLY, and acetone with their values varying from 41.2 to 46.8 while the lower efficiencies were observed among the other five carbonyl derivatives with their values in the range of 6.8– 21.5. From the equation of E1 the extraction efficiency is positively correlated to the octanol/water partitioning coefficient (*K*_o/w_) of the analyte. The order of *K*_o/w_ values of above nine carbonyls is acetone (log *_K_*_o/w_ = –0.48) < isobutenal (log *_K_*_o/w_ = 0.10) < butenone (log *_K_*_o/w_ = 0.26) < formaldehyde (log *_K_*_o/w_ = 0.35) < 2‐hyroxy ethanal < GLY < methyl glyoxal < ethanal (log *_K_*_o/w_ = 0.52) < octanal (log *_K_*_o/w_ = 1.85) 27. Thus, it is reasonable to consider that the *K*_o/w_ values of their corresponding derivatives follow the similar order as the above mentioned due to the lack of *K*_o/w_ values of the derivative. The *K*_o/w_ values can be used to explain the reasons for the higher preconcentration efficiencies of octanal, GLY, and MGLY and the lower efficiencies of isobutenal, butenone, formaldehyde, and 2‐hydroxy ethanal, respectively. However, *K*_o/w_ cannot explain the reason for the high ratio of acetone derivative and low ratio of acetaldehyde.

### 3.6 Calibrations and detection limits

The parameters of calibration curves for 29 carbonyls are listed in Table 2. It is found that the slope values of those calibration curves follow the order of formaldehyde > dicarbonyls > aldehydes > aromatic aldehydes > ketones. The sensitivity of the SBSE method was evaluated in terms of LOD. The LODs of SBSE to 29 carbonyls vary from 0.02 to 0.24 μg/L (Table 2). The comparisons between the SBSE derivatization technique and other previously reported methods for measuring aqueous carbonyls in terms of LOD are listed in Table 4. The LODs of this SBSE technique for carbonyl measurements are lower than those of GC–ECD 28, and SPME–HPLC–UV 29, by 10 to 100 times and close to those of SBSE–HPLC–MS 30 and solvent extraction GC–ECD techniques 31.

**Table 4:** Comparisons of pre‐treatment technique, total analysis time, sample volume, and limit of detection (LOD) of SBSE‐GC‐MS with other analytical techniques on carbonyl determinations in rainwater

	SBSE‐GC‐MS	GC‐ECD[28]	Solvent Extraction GC‐ECD[31]	SPME‐HPLC‐UV[29]	SBSE‐HPLC‐MS[11]
Pre‐treatment Technique	SBSE	no	no	SPME	SPME
Total Analysis Time a) (min)	140	175	98.5	90	1440
Sample Volume (mL)	100	50	50	5	5‐70
LOD of Carbonyls (ng/L)					
1 Formaldehyde	14	2700	7.6	500	–
2 Ethanal	31	670	16.8	90	–
3 Acetone	57	–b)	22.4	–	–
4 Propanal	27	430	20.8	360	–
5 Propenal	33	–	–	–	–
6 Butanal	28	270	10.6	–	–
7 Isobutenal	236	–	–	–	–
8 Butenone	189	–	–	–	–
9 2‐Hydroxy ethanal	178	–	–	–	–
10 2‐Pentanone	76	–	–	–	–
11 3‐Methylbutanal	57	–	–	–	–
12 2‐Butenal	42	–	–	–	–
13 Pentanal	21	740	10.2	230	–
14 2‐Hexanone	112	–	–	–	–
15 Hydroxyacetone	122	–	–	–	–
16 Pentane‐2,4‐dione	236	–	–	–	–
17 Hexanal	32	1290	14.2	–	–
18 2‐Hexenal	47	–	–	–	–
19 Heptanal	31	630	27.9	–	–
20 2‐Octanone	198	–	–	–	–
21 Octanal	31	400	6.9	–	–
22 4‐Fluorobenzaldehyde	59	–	–	–	–
23 Benzaldehyde	46	910	10.9	–	–
24 o‐Tolualdehyde	42	–	–	–	–
25 m‐Tolualdehyde	67	–	–	–	–
26 *p*‐Tolualdehyde	84	–	–	–	–
27 2,4‐Bimethylbenzaldehyde	72	–	–	–	–
28 Glyoxal	16	2220	17.3	–	16
29 Methylglyoxal	18	750	4.95	–	14

a) Total analysis time includes derivatisation time, extraction time, desorption time, GC or HPLC analysis time.

b) No data available.

The matrix effect is considered as a major limitation to SBSE technique for some complex samples such as environmental samples, biological fluids, or foods 25. Rainwater is somewhat simpler in chemical composition compared with above examples. In this study, the matrix effects of the chemicals including K^+^, Na^+^, NH_4_^+^, Ca^2+^, Mg^2+^, Cl^−^, NO_3_^−^, SO_4_^2–^ and humic acid (1–2 mg/L) were studied. No significant influence was found on the described SBSE approach to carbonyl measurements. The recoveries by the SBSE technique for aqueous carbonyls with above chemical interferences varied from 79 to 105% with most data close to 90% (Table 2), which implied the SBSE derivatization technique can determine most amounts of carbonyls in aqueous solution even with the presence of common matrix compounds.

The repeatability and reproducibility of SBSE technique were evaluated by determining five ultrapure water samples. The repeatability varies from 4.8 to 14.6% with most RSD < 10%. The reproducibility of the RSD values varied from 7.6 to 14.7% and for some unsaturated carbonyls had RSDs >10% such as isobutenal, butenone, hexenal, and some multifunctional carbonyls including hydroxyactone, GLY, and MGLY.

### 3.7 Application in rainwater measurement

**Figure 5:**
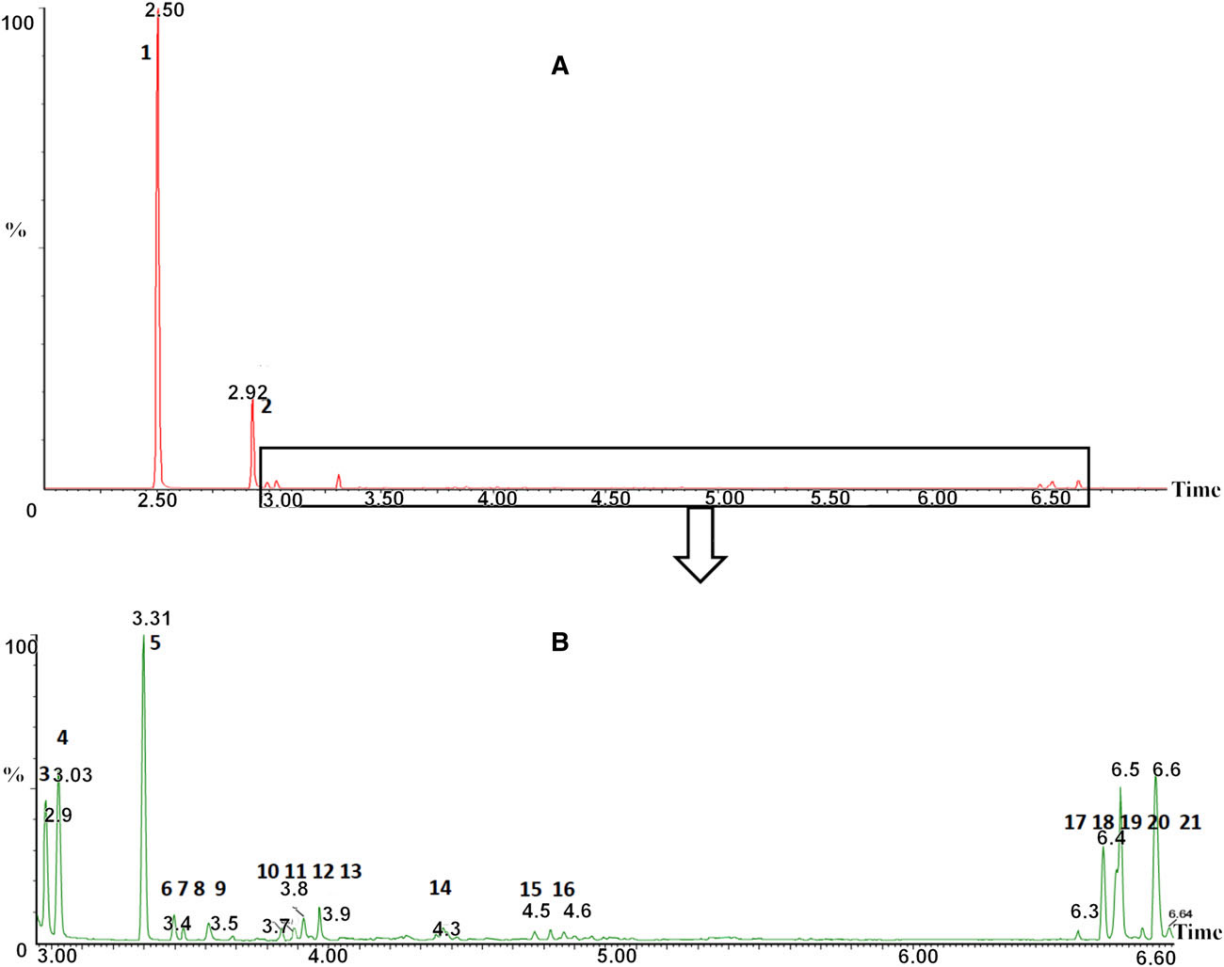
Carbonyls absorbed from rainwater (30 September, 2014) by SBSE. Panel A: The solutions from the desorption solvent. Panel B is the enlargement of the partial panel A in the range of RT of 3.00 min to 6.65 min. Peaks in GC chromatograms are formaldehyde (peak 1), PFBHA (peak 2), ethanal (peaks 3 and 4), acetone (peak 5), propanal (peaks 6 and 7), propenal (peak 8), butanal (peak 9), isobutenal (peak 10), butanone (peak 11), 2‐hydroxy ethanol (peaks 12 and 13), pentanal (peak 14), hexanal (peaks 15 and 16), GLY (peaks 17, 18, and 19), and MGLY (peaks 20 and 21), respectively

SBSE derivatization technique was deployed to determine carbonyls in rainwater samples collected from July to November 2014 in York, UK. The GC chromatograms (Fig. 5) of rainwater samples show 20 carbonyl species present in the rainwater samples. The carbonyl concentrations in rainwater measured by SBSE technique are listed in Table 5. The carbonyl concentrations appeared highest in July rainwater with total concentration of 753 μg/L and decreased from August, September, November, and October, successively. The largest precipitation of carbonyls in rainwater occurred in August with a value of 22 455 μg/m^2^ and the lowest precipitation was 6011 μg/m^2^ in November. The concentration of carbonyl compounds in rainwater depends on their ambient gaseous concentrations, their water solubility and Henry's constant, the frequency of rain events, and the rainfall intensity 28. Some carbonyls such as formaldehyde, ethanal, and acetone are predominant carbonyl species in ambient air and their concentrations in rainwater were higher than those of other carbonyls, which is consistent to the results in previous studies 32. Some specific carbonyls including butenal, butenone, GLY, MGLY, hydroxyactone in ambient air were much higher in the rainwater of July and August, which may arise from the photooxidation of atmospheric isoprene from biogenic emissions 7. In July and August, the deciduous plants near the sampling site were in the full growth and their period of peak isoprene emission. Therefore, it is reasonable to consider the isoprene photooxidation made a greater contribution to ambient gaseous carbonyls and aqueous carbonyls in rainwater in July and August. GLY and MGLY are highly water soluble and can form SOAs through uptake into the aqueous phase of a cloud droplet, which significantly impacts climate and air quality 33. In August the wet deposition of GLY and methylgloxal were ten times higher than in November, which implies the two dicarbonyls may participate into the formation of cloud water.

**Table 5:** Carbonyl concentrations and carbonyl deposition in rainwater collected from July to November 2014 in York, UK based on SBSE technique on aqueous carbonyl analysis

		Concentration (μg/L)					Deposition (μg/m^2^)				
No.	Carbonyls	Jul.	Aug.	Sep.	Oct.	Nov.	Jul.	Aug.	Sep.	Oct.	Nov.
1	Formaldehyde	161	88.2	67.6	42.1	74.5	2222	4463	1784	1414	1676
2	Ethanal	137	84.4	44.2	22.7	49.8	1890	4271	1167	763	1120
3	Acetone	104	48	32.4	14.7	42.6	1435	2429	855	494	958
4	Propanal	38.9	25.6	22.4	11.5	30.1	536	1295	591	386	677
5	Propenal	19.5	10.4	12.4	6.4	2.4	269	526	327	215	54
6	Butanal	22.1	14.6	13.7	10.3	10.8	305	739	362	346	243
7	Isobutenal	6.5	4.5	3.4	0.5	0.6	89.7	228	89.7	16.8	13.5
8	Butenone	12.3	5.6	3.1	Nda)	Nd	169	283	81.8	Nd	Nd
9	2‐Hydroxy ethanal	48.6	32.7	24.6	10.3	5.9	670	1655	649	346	132
10	2‐Pentanone	Nd	Nd	Nd	Nd	Nd	Nd	Nd	Nd	Nd	Nd
11	3‐Methylbutanal	6.2	4.5	1.2	2.3	4.2	85.6	228	32	77.2	94.5
12	2‐Butenal	7.4	4.1	4.3	4.2	8.1	102	207	113	141	182
13	Pentanal	24.5	15.4	12.6	10.5	3.5	338	779	333	353	78.8
14	2‐Hexanone	5.7	5.6	1.4	2.1	2.4	78.7	283	37	70.5	54
15	Hydroxyacetone	18.5	15.1	9.4	6.2	1.3	255	764	248	208	29.2
16	Pentane‐2,4‐dione	Nd	Nd	Nd	Nd	Nd	Nd	Nd	Nd	Nd	Nd
17	Hexanal	25.1	11.4	10.5	8.5	2.4	346	577	277	285	54
18	2‐Hexenal	4.3	3.5	Nd	Nd	Nd	59.3	177	Nd	Nd	Nd
19	Heptanal	25.4	18.6	10.5	8.5	3.5	350	941	277	285	78.8
20	2‐Octanone	Nd	Nd	Nd	Nd	Nd	Nd	Nd	Nd	Nd	Nd
21	Octanal	19.5	10.6	8.4	6.3	4.5	269	536	222	212	101
22	4‐Fluorobenzaldehyde	Nd	Nd	Nd	Nd	Nd	Nd	Nd	Nd	Nd	Nd
23	Benzaldehyde	Nd	Nd	Nd	10.3	11.3	Nd	Nd	Nd	346	254
24	*o‐*Tolualdehyde	Nd	Nd	Nd	Nd	Nd	Nd	Nd	Nd	Nd	Nd
25	*m‐*Tolualdehyde	Nd	Nd	Nd	Nd	Nd	Nd	Nd	Nd	Nd	Nd
26	*p*‐Tolualdehyde	Nd	Nd	Nd	Nd	Nd	Nd	Nd	Nd	Nd	Nd
27	2,4‐Bimethylbenzaldehyde	Nd	Nd	Nd	Nd	Nd	Nd	Nd	Nd	Nd	Nd
28	GLY	40.7	27.6	16.8	6.5	5.6	562	1396	443	218	126
29	MGLY	26.4	13.4	8.5	3.6	3.8	364	678	224	120	85.5
30	Total carbonyls	753	444	307	187	267	10 395	22 455	8112	6296	6011

a) Not detect.

## 4 CONCLUDING REMARKS

In this study, a SBSE–GC–MS method was successfully developed to simultaneously extract and analyze 29 species of carbonyls from aqueous samples. Carbonyls were chemically derivatized to their nonpolar oxime derivatives by PFBHA and extracted from aqueous matrices by the PDMS coating on stir bars. The preconcentration efficiencies of SBSE for most carbonyls can reach factors of 40 when the carbonyls were extracted from 100 mL sample and desorbed into 2 mL solvent solution. SBSE technique was employed for the carbonyl analysis in rainwater and the extraction efficiencies were > 85% for 20 carbonyl species. The SBSE–GC–MS technique has proved to be both sensitive and specific for the quantification of trace carbonyls in rainwater samples with complicated matrices.
